# SIRT3 deficiency-induced mitochondrial dysfunction and inflammasome formation in the brain

**DOI:** 10.1038/s41598-018-35890-7

**Published:** 2018-12-03

**Authors:** Alpna Tyagi, Christy U Nguyen, Thomas Chong, Cole R Michel, Kristofer S. Fritz, Nichole Reisdorph, Leslie Knaub, Jane E. B. Reusch, Subbiah Pugazhenthi

**Affiliations:** 1 Department of Medicine, Rocky Mountain Regional VA Medical Center, Aurora, CO USA; 2 School of Medicine, University of Colorado-Anschutz Medical Campus, Aurora, CO USA; 3 Department of Pharmaceutical Sciences, University of Colorado-Anschutz Medical Campus, Aurora, CO USA

## Abstract

SIRT3, the primary mitochondrial deacetylase, plays a significant role in enhancing the function of mitochondrial proteins. Downregulation of SIRT3 is a key component of metabolic syndrome, a precondition for obesity, diabetes and cardiovascular diseases. In this study, we examined the effects of brain mitochondrial protein hyperacetylation in western diet-fed Sirt3^−/−^ mice, a model for metabolic syndrome. Brain mitochondrial proteins were hyperacetylated, following western diet feeding and Sirt3 deletion. To identity these hyperacetylated proteins, we performed a comprehensive acetylome analysis by label-free tandem mass spectrometry. Gene ontology pathway analysis revealed Sirt3 deletion-mediated downregulation of enzymes in several metabolic pathways, including fatty acid oxidation and tricarboxylic acid cycle. Mitochondrial respiration was impaired at multiple states, along with lower levels of mitochondrial fission proteins Mfn1 and Mfn2. Cleavage of procaspase-1 suggested inflammasome formation. Assembly of inflammasomes with caspase-1 and NLRP3 was detected as shown by proximity ligation assay. Markers of neuroinflammation including microgliosis and elevated brain IL-1β expression were also observed. Importantly, these findings were further exacerbated in Sirt3^−/−^ mice when fed a calorie-rich western diet. The observations of this study suggest that SIRT3 deficiency-induced brain mitochondrial dysfunction and neuroinflammation in metabolic syndrome may play a role in late-life cognitive decline.

## Introduction

Sirtuins are a family of seven proteins with NAD^+^-dependent deacetylase activity. These conserved proteins play important roles in the regulation of metabolism, inflammation and longevity^1,2^. They are characterized by distinct subcellular localization. For example, SIRT1, SIRT6 and SIRT 7 are localized in the nucleus; SIRT2 is cytosolic; and SIRT3-5 are mitochondrial sirtuins. Among the three mitochondrial sirtuins, SIRT3 is the primary deacetylase. While SIRT5 regulates malonylation and succinylation, SIRT4 with lysine deacylase activity regulates leucine metabolism and insulin secretion^3^. Mitochondrial protein lysine acetylation plays a key role in metabolism, ATP production and antioxidant defense. Cellular NAD levels are critical regulators of SIRT3 activity^4^. Whereas calorie restriction and fasting increase NAD levels, high fat diet-feeding significantly reduces the NAD levels. Thus, calorie intake regulates mitochondrial function through SIRT3 activation.

Mice with global Sirt3 deletion are viable but display dysfunction in multiple tissues, especially under conditions of stress. For example, cardiac recovery is impaired following myocardial ischemia^5^ and cisplatin-induced acute kidney injury is exacerbated^6^ in Sirt3^−/−^ mice. Impairment of Insulin signaling in the skeletal muscles of Sirt3^−/−^ mice^7^ is further exacerbated, following high fat feeding^8^. Mitochondrial fatty acid oxidation is decreased in the liver of Sirt3^−/−^ mice^9^. However, liver and muscle-specific Sirt3 deletion does not recapitulate the metabolic abnormalities observed in Sirt3^−/−^ mice^10^. Hirschey *et al*. (2011) demonstrated the acceleration of metabolic syndrome (MetS), characterized by obesity, insulin resistance and hyperlipidemia in Sirt3^−/−^ mice, following high-fat diet feeding^11^. Cheng *et al*. (2016) have demonstrated by *in vivo* and *in vitro* studies that SIRT3 mediates adaptive response to oxidative and excitatory stress in the neurons of the brain^12^.

Protein acetylation is recognized as an important metabolic regulatory mechanism in the mitochondria^13^. Acetylome analysis of liver mitochondria from Sirt3^−/−^ mice has revealed SIRT3-mediated acetylation on multiple proteins, often at multiple sites, across several metabolic pathways including fatty acid oxidation, ketogenesis, amino acid catabolism, and tricarboxylic acid (TCA) cycle, as well as other key regulatory proteins in mitochondria^14^. Another acetyl proteomic study showed that calorie restriction-mediated deacetylation of liver mitochondrial proteins is regulated by SIRT3^15^. Dittenhafer-Reed *et al*. (2015) examined the acetylome of multiple tissues of Sirt3^−/−^ mice and identified the mitochondrial pathways common to these tissues^16^. This study also identified the differences between fuel-producing and fuel utilizing tissues in terms of SIRT3 targets. Brain accounts for 20% of total body energy consumption although it is only 2% of body weight. Substrates needed for the brain are generated by the peripheral tissues. Therefore, dysregulation of brain energy metabolism can be expected in MetS. Hyperacetylated mitochondrial proteins under conditions of calorie overload have not been examined in the brain.

MetS, the precondition for obesity and diabetes is caused by genetic predisposition and life style changes. The increase in the rate of obesity globally is linked to the consumption of western diet, rich in simple sugars and saturated fat. While the research in MetS has generally remained in the domain of peripheral tissues, the effects of MetS in the CNS is also being recognized. MetS has been shown to be associated with late-life dementia^17,18^. Western diet consumption is reported to cause hippocampal dysfunction^19,20^. Several mechanisms may be involved in MetS-mediated cognitive decline^21^. For example, elevated saturated fatty acids can be taken up by the brain and trigger inflammation through activation of toll-like receptors^22,23^. Insulin resistance in MetS is another factor that can cause cognitive dysfunction^24^. Furthermore, macrophages that infiltrate the visceral fat in MetS produce proinflammatory cytokines which can cross the blood brain barrier (BBB), leading to chronic inflammation in the CNS^25^. Mitochondrial injury leads to formation of inflammasome^26–28^, a multiprotein cytosolic complex that is generated in response to infection, cellular damage, and metabolic dysregulation^29^. Inflammasome activates the proinflammatory caspase-1 which proteolytically cleaves and secretes the cytokines IL-1β and IL-18^30^. Mitochondrial DNA released into the cytosol following injury^31^, damage-associated molecular patterns, such as the high mobility group proteins HMGB1 and ATP^32–34^ are also known to induce inflammasomes. Thus, in addition to the circulating factors playing a role in neuroinflammation, inflammatory pathways can be triggered within the brain because of metabolic stress.

Although the role of SIRT3 in the mitochondrial function of peripheral tissues has been examined extensively, Sirt3 function in the CNS is an understudied area of research. It is generally believed that brain is protected from the deleterious effects of diet-induced MetS and the observed CNS effects are secondary to systemic changes. However, MetS can induce mitochondrial injury in the brain itself which can trigger the neuroinflammatory pathway. Therefore, we hypothesize that SIRT3 deficiency-mediated mitochondrial dysfunction leads to inflammasome formation in the brain of MetS and thereby setting the stage for chronic CNS inflammation, one of the causes of cognitive decline. Therefore, the objective of this study was to identify the downregulated SIRT3 targets in the brain, evaluate mitochondrial dysfunction and determine if it leads to inflammasome formation in western diet-fed, Sirt3^−/−^ mice, a model of MetS.

## Results

### Acceleration of metabolic syndrome (MetS) in western diet-fed Sirt3^−/−^ mice

**Figure 1:**
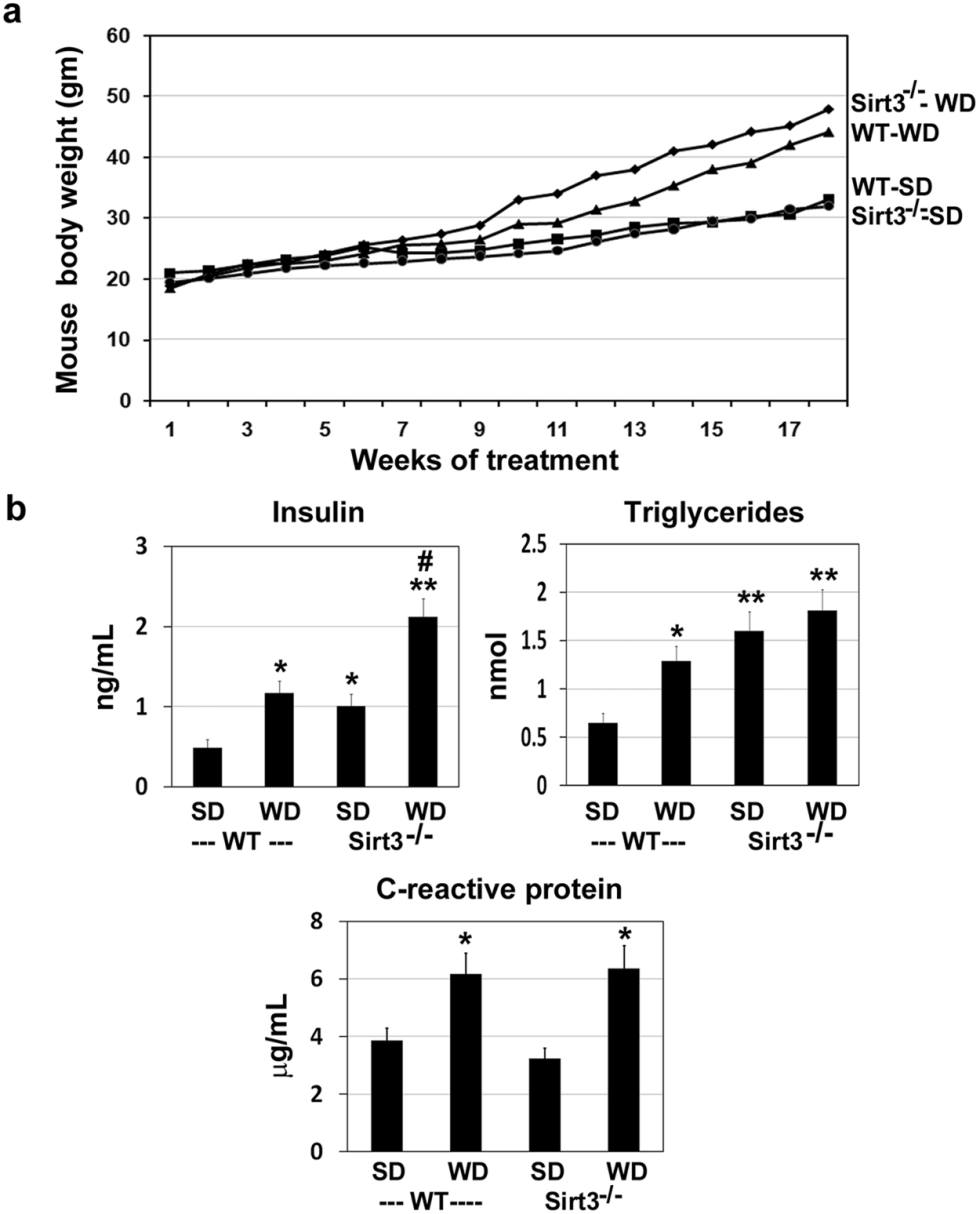
Western diet feeding leads to weight gain, insulin resistance and inflammation in wild type and Sirt3^−/−^ mice. Two month-old male wild type and Sirt3^−/−^ mice were fed standard diet (SD) or western diet (WD) for 4 months. (**a**) The mouse body weight gain was monitored during the course of feeding different diets. (**b**) Insulin, triglycerides and C-reactive protein (CRP) levels were measured in the plasma samples, using standard kits at the end of the study. Values are mean ± SE of 6 mice in each group. *P < 0.01; **P < 0.001, compared to wild type mice on standard diet. ^#^*P* < 0.001 vs Sirt3^−/−^ mice on standard diet.

MetS, a risk factor for obesity, diabetes and cardiovascular diseases, is caused by genetic factors and life style changes. Feeding calorie-rich diet to Sirt3^−/−^ mice has been shown to induce MetS^11^. Many previous studies have used high fat diet whereas in this study, we fed the mice with western diet, rich in saturated fat and simple sugars, to recapitulate a model for the current life style. Diet-induced obesity was evident by the significant weight gain of 33% and 50% in wild type and Sirt3^−/−^ mice, respectively (Fig. 1a). Measurement of plasma insulin showed significant diet-induced hyperinsulinemia (2.4-fold increase) in wild type mice. In Sirt3^−/−^ mice, insulin resistance was evident with standard diet itself as shown by 2.1-fold higher plasma insulin levels whereas in western diet-fed Sirt3^−/−^ mice, insulin levels were 4.3-fold higher (Fig. 1b). In the case of plasma triglyceride levels, 2-fold higher levels were observed in wild type mice, following western diet feeding and 2.4 and 2.8-fold higher levels were detected in Sirt3^−/−^ mice with standard and western diets respectively (Fig. 1b). Western diet also significantly increased the circulating inflammatory marker, C-reactive protein (CRP), in both wild type and Sirt3^−/−^ mice as compared to their respective controls fed standard diet (Fig. 1b). These general parameters are like in previous reports, suggesting that diet-induced weight gain, insulin resistance and hypertriglyceridemia in Sirt3^−/−^ mice, as an experimental model to study MetS. While the previous studies have examined peripheral tissues, including liver, muscle and heart in these mice, the current study focused on the brain.

### Hyperacetylation of mitochondrial proteins in Sirt3^−/−^ mouse brain

**Figure 2:**
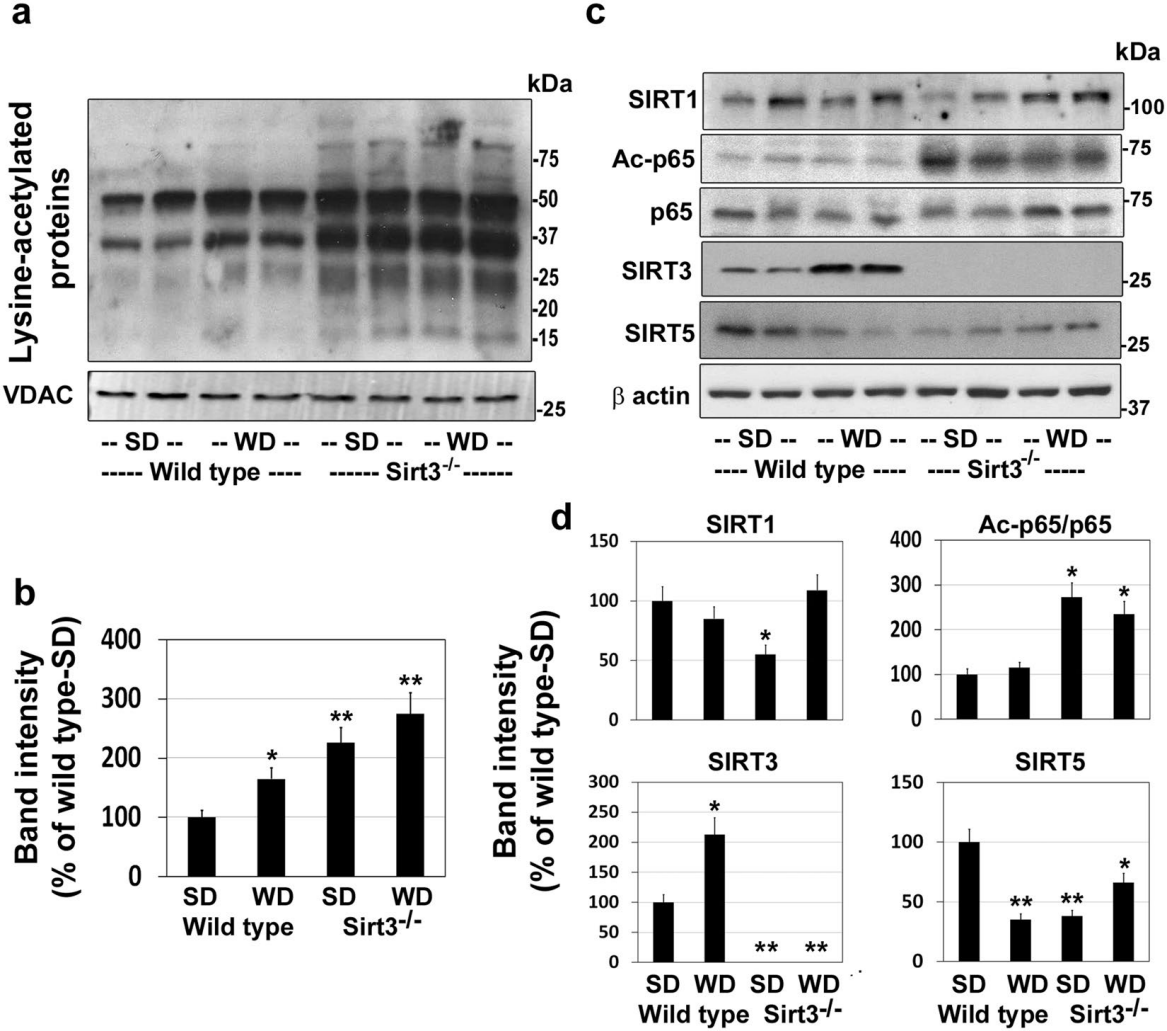
Hyperacetylation of brain mitochondrial proteins in western diet-fed mice. Male wild type and Sirt3^−/−^ mice were fed standard diet (SD) or western diet (WD) for 4 months. (**a**) Western blot analysis was performed with the brain mitochondrial protein extracts, using a pan anti-lysine antibody. The blots were reprobed for VDAC, a marker for mitochondria. Blots for two representative samples in each group are presented. (**b**) Hyperacetylated protein bands were scanned and the total band intensities were corrected for VDAC levels. (**c**) The levels SIRT1, Ac-p65, p65, SIRT3 and SIRT5 were examined by western blotting. (**d**) Densitometric analysis of band intensity for each blot was performed and corrected for β actin levels. Values are mean ± SE of six samples. *P < 0.01; **P < 0.001 vs wild type mice on standard diet.

Acetylation is an important posttranslational modification that plays a critical role in metabolic regulation^35^. Because SIRT3 is the primary mitochondrial deacetylase, we examined the total protein acetylation in isolated mitochondria from the four groups of mice by western blot analysis, using a pan acetyl lysine antibody. As expected, calorie overload as well as Sirt3 deletion resulted in significant hyperacetylation of multiple mitochondrial proteins (Fig. 2a). Quantitation of cumulative band intensities revealed 65–175% higher protein lysine acetylation in mitochondria with maximum increase being observed in Sirt3^−/−^ mice fed on western diet (Fig. 2b). Feeding a diet rich in fat content results in elevation of the steady-state levels of acetyl CoA which causes protein modification at lysine residues. To determine if Sirt3 deletion and western diet feeding affects the levels of other sirtuins, we performed western blot analysis of brain samples. The levels of SIRT1, another well investigated sirtuin, was lower in western diet-fed wild type mice and standard diet-fed Sirt3^−/−^ mice, following calorie excess (Fig. 2c). SIRT1 downregulates the proinflammatory transcription factor p65 by deacetylation at lysine 310. The levels of acetylated p65 were 173% and 135% higher in Sirt3^−/−^ mice fed on standard and western diet respectively, suggesting decreased SIRT1 activity. As expected, SIRT3 protein was absent in Sirt3^−/−^ mouse brain, confirming the deletion of Sirt3 gene. Interestingly, a 2-fold higher SIRT3 levels were observed in wild type mice after feeding western diet which appears to be a compensatory response to calorie excess. However, despite the elevated SIRT3 protein levels (Fig. 2c), a 65% higher protein acetylation was observed in this group (Fig. 2b), probably because of decreased activity. Calorie overload is expected to deplete NAD^+^, a cofactor for SIRT3, the primary mitochondrial deacetylase. A previous study has reported a similar higher SIRT3 levels initially, followed by decrease in this protein after chronic high fat feeding^36^. The levels of SIRT5, another mitochondrial deacetylase, were also lower by 65% and 62% in response to western diet and Sirt3 deletion respectively. The levels of other sirtuins did not change (results not shown).

### Identification of hyperacetylated brain mitochondrial proteins by acetylome analysis

**Figure 3:**
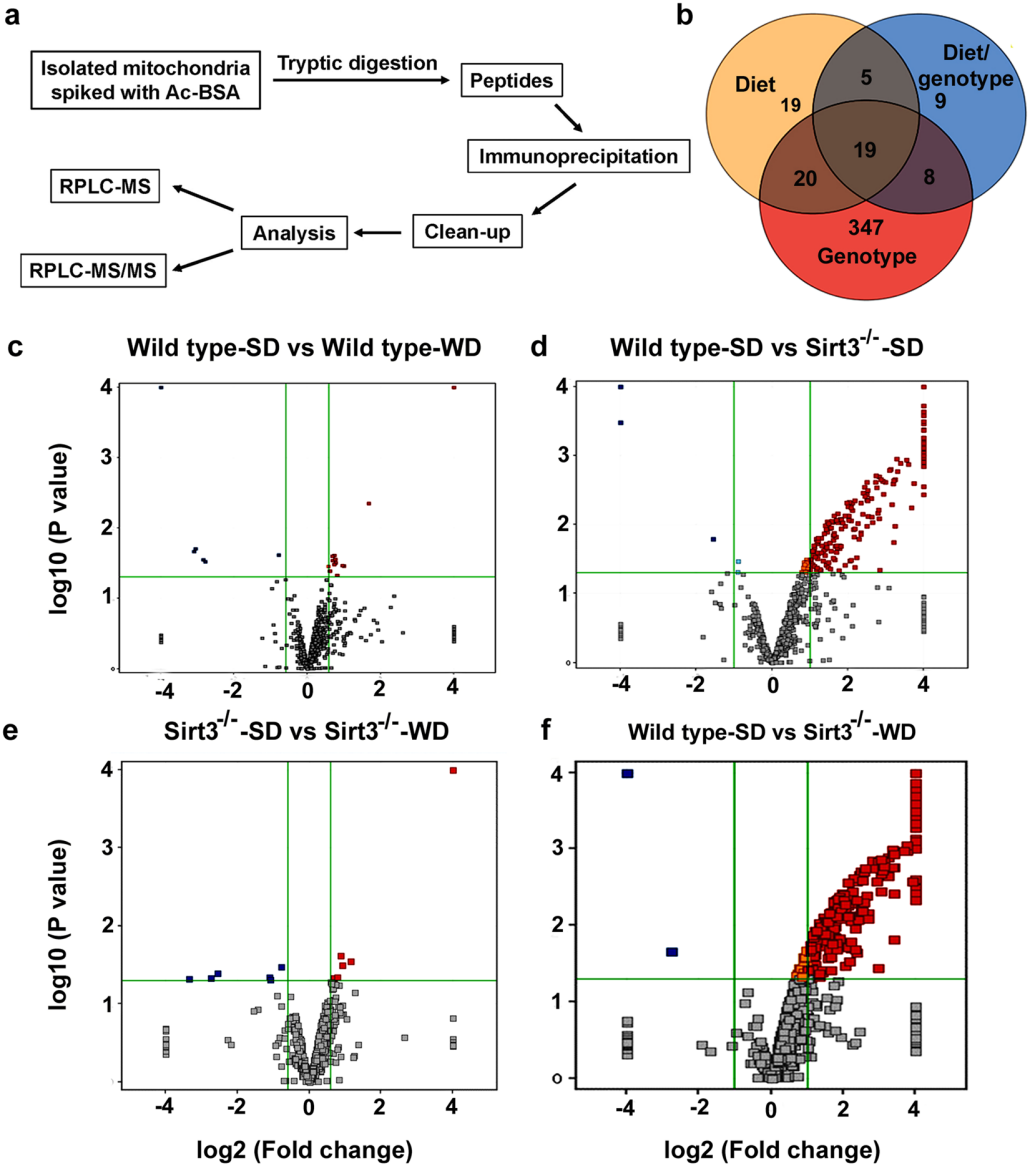
Mitochondrial protein acetylome analysis. (**a**) A comprehensive acetylome analysis was performed with six samples for each group as described in the flow chart. (**b**) The Venn diagram shows the effects of genotype, diet and combination on the number of hyperacetylated peptides. The fold changes in hyperacetylated proteins are presented in the Volcano plots for the effects of diet (**c**,**e**), genotype (**d**) and the combination (**f**).

To determine the identity of hyperacetylated mitochondrial proteins, we performed a comprehensive acetylome analysis. The schematic diagram (Fig. 3a) describes the sequential steps that were performed. Trypsin-digested mitochondrial proteins were first separated by liquid chromatography and quantitated (MS-only mode). Following data acquisition in MS/MS mode, the database search program, SpectrumMill was used to identify the acetylated peptides and proteins. A total of 993 acetylated peptides were identified. Venn diagram (Fig. 3b) shows the number of entities modified in response to diet, genotype and the combination. Volcano plots (Fig. 3c–f) show the number of acetylated peptides with a fold change of >2.0 and a P value of <0.05, following western diet feeding and Sirt3 deletion. In wild type mice, calorie overload resulted in 23 acetyl-lysine peptides being elevated whereas Sirt3 deletion resulted in the hyperacetylation of 248 peptides. The combination showed significant elevation in 265 acetylated peptides. Overall, the western diet-fed Sirt3^−/−^ mouse brain samples showed maximum effects in terms of mitochondrial protein acetylation (Fig. 3f). In this group, 103 mitochondrial proteins were identified with significant hyperacetylation with many SIRT3 targets being hyperacetylated at multiple sites. These proteins were subjected to Gene ontology (GO)/pathway analysis. Table 1 summarizes the biochemical pathways that are affected by western diet feeding and by Sirt3 deletion. The diet had smaller effects on the hyperacetylation of mitochondrial proteins whereas Sirt3 deletion caused major changes by downregulation of metabolic enzymes. Because the combination of western diet and Sirt3 deletion, a model for metabolic syndrome, showed maximum changes, Tables 2 and 3 lists the individual proteins under different pathways. Taking the acetylation levels in wild type mice on standard diet as 1, we have presented the fold increases. The major pathways affected were electron transport chain, fatty acid oxidation, TCA cycle and redox pathway in which mitochondria play a central role.

**Table 1:** Major biochemical pathways of hyperacetylated mitochondrial proteins in different groups.

Pathway	Number of proteins	Average fold increase
**Wild type-SD vs Wild type-WD**		
TCA cycle/Glycolysis	2	1.57
Electron transport chain	—	—
Fatty acid oxidation	2	1.63
Anti-oxidant defense	1	1.94
**Wild type-SD vs Sirt3** ^**−/−**^ **-SD**		
TCA cycle/Glycolysis	14	9.84
Electron transport chain	13	7.6
Fatty acid oxidation	12	15.74
Anti-oxidant defense	3	5.96
**Sirt3** ^**−/−**^ **-SD vs Sirt3** ^**−/−**^ **-WD**		
TCA cycle/Glycolysis	1	2.22
Electron transport chain	2	1.67
Fatty acid oxidation	—	—
Anti-oxidant defense	—	—
**Sirt3** ^**−/−**^ **-SD vs Sirt3** ^**−/−**^ **-WD**		
TCA cycle/Glycolysis	1	2.22
Electron transport chain	2	1.67
Fatty acid oxidation	—	—
Anti-oxidant defense	—	—
**Wild type-SD vs Sirt3** ^**−/−**^ **-WD**		
TCA cycle/Glycolysis	18	9.77
Electron transport chain	20	8.90
Fatty acid oxidation	20	6.01
Anti-oxidant defense	7	4.77

Following acetylome analysis, identified acetylated proteins were subjected to Gene ontology (GO)/pathway analysis. A summary of selected biochemical pathways that were affected by western diet feeding and Sirt3 deletion are presented.

**Table 2:** List of hyperacetylated mitochondrial proteins in electron transport chain and fatty acid oxidation by a combination of western diet and Sirt3 deletion.

	Electron Transport chain	Fold ↑
1	NADH dehydrogenase [ubiquinone] 1 alpha subcomplex subunit 9, mitochondrial, Ndufa9	3.6
2	ADP/ATP translocase 1, Slc25a4	31.7
3	ATP synthase subunit O, mitochondrial, Atp5o	6.8
4	NADH dehydrogenase [ubiquinone] flavoprotein 2, mitochondrial, Ndufv2	2.6
5	ATP synthase protein 8, Mtatp8	2.1
6	Dihydrolipoyl dehydrogenase, mitochondrial, Dld	4.3
7	ATP synthase subunit epsilon, mitochondrial, Atp5e	20.6
8	NADH dehydrogenase [ubiquinone] 1 alpha subcomplex subunit 7, Ndufa7	8.3
9	AFG3-like protein 1, Afg3l1	10.5
10	Dihydrolipoyllysine-residue acetyltransferase component of pyruvate dehydro complex	17.6
11	Mitochondrial glutamate carrier 1, Slc25a22	5.1
12	NADH dehydrogenase [ubiquinone] 1 alpha subcomplex subunit 2, Ndufa2	3.6
13	Pyruvate dehydrogenase protein X component, mitochondrial, Pdhx	10.4
	**Fatty acid oxidation**	
1	Trifunctional enzyme subunit alpha, mitochondrial, Hadha	14.1
2	ATP synthase subunit beta, mitochondrial, Atp5b	2.8
3	ATP synthase subunit alpha, mitochondrial, Atp5a1	3.9
4	Acyl-coenzyme A thioesterase THEM4, Them4	3.0
5	Hydroxyacyl-coenzyme A dehydrogenase, mitochondrial, Hadh	8.4
6	3-ketoacyl-CoA thiolase, mitochondrial, Acaa2	5.4
7	Methylglutaconyl-CoA hydratase, mitochondrial, Auh	4.5
8	3-oxoacyl-[acyl-carrier-protein] synthase, mitochondrial, Oxsm	3.0
9	Acyl-CoA synthetase family member 2, mitochondrial, Acsf2	4.0
10	Short/branched chain specific acyl-CoA dehydrogenase, mitochondrial, Acadsb	2.3
11	Acetyl-CoA acetyltransferase, mitochondrial, Acat1	10.4
12	Adenylate kinase 4, mitochondrial, Ak4	10.6

The fold increase in hyperacetylation of mitochondrial proteins in electron transport chain and fatty acid oxidation by a combination of Sirt3 deletion and western diet feeding are listed, taking the levels in wild type mice on standard diet as 1.

**Table 3:** List of hyperacetylated mitochondrial proteins in TCA cycle and redox pathway by a combination of western diet and Sirt3 deletion.

	TCA Cycle	Fold ↑
1	Pyruvate dehydrogenase E1 component subunit beta, mitochondrial, Pdhb	9.3
2	Pyruvate dehydrogenase E1 component subunit alpha, testis-specific form, mitochondrial	5.8
3	Isocitrate dehydrogenase [NADP], mitochondrial, Idh2	2.1
4	Malate dehydrogenase, mitochondrial, Mdh2	34.3
5	Succinyl-CoA ligase [ADP-forming] subunit beta, mitochondrial, Sucla2	7.5
6	Fumarate hydratase, mitochondrial, Fh	7.4
7	Succinyl-CoA ligase [ADP/GDP-forming] subunit alpha, mitochondrial, Suclg1	7.4
8	Citrate synthase, mitochondrial, Cs	5.2
9	Succinate dehydrogenase [ubiquinone] flavoprotein subunit, mitochondrial, Sdha	29.4
10	Succinate dehydrogenase [ubiquinone] iron-sulfur subunit, mitochondrial, Sdhb	2.3
11	Aconitate hydratase, mitochondrial, Aco2	7.0
12	Isocitrate dehydrogenase [NAD] subunit alpha, mitochondrial, Idh3a	2.8
13	Isocitrate dehydrogenase [NAD] subunit gamma 1, mitochondrial, Idh3g	2.7
14	Pyruvate dehydrogenase E1 component subunit α, somatic form, mitochondrial, Pdha1	7.2
15	Glutamate dehydrogenase 1, mitochondrial, Glud1	5.5
16	Succinate-semialdehyde dehydrogenase, mitochondrial, Aldh5a1	8.7
17	Pyruvate dehydrogenase protein X component, mitochondrial, Pdhx	10.4
18	Mitochondrial glutamate carrier 1, Slc25a22	5.1
	**Redox pathway**	
1	Peroxiredoxin-5, mitochondrial, Prdx5	5.0
2	Superoxide dismutase [Mn], mitochondrial, Sod2	3.1
3	NAD(P) transhydrogenase, mitochondrial, Nnt	6.0
4	Peptidyl-prolyl cis-trans isomerase F, mitochondrial,Ppif	5.1

The fold increase in hyperacetylation of mitochondrial proteins in TCA cycle and redox pathway by a combination of Sirt3 deletion and western diet feeding are listed, taking the levels in wild type mice on standard diet as 1.

### Lower levels of mitochondrial fission/fusion proteins in Sirt3^−/−^ mouse brain

**Figure 4:**
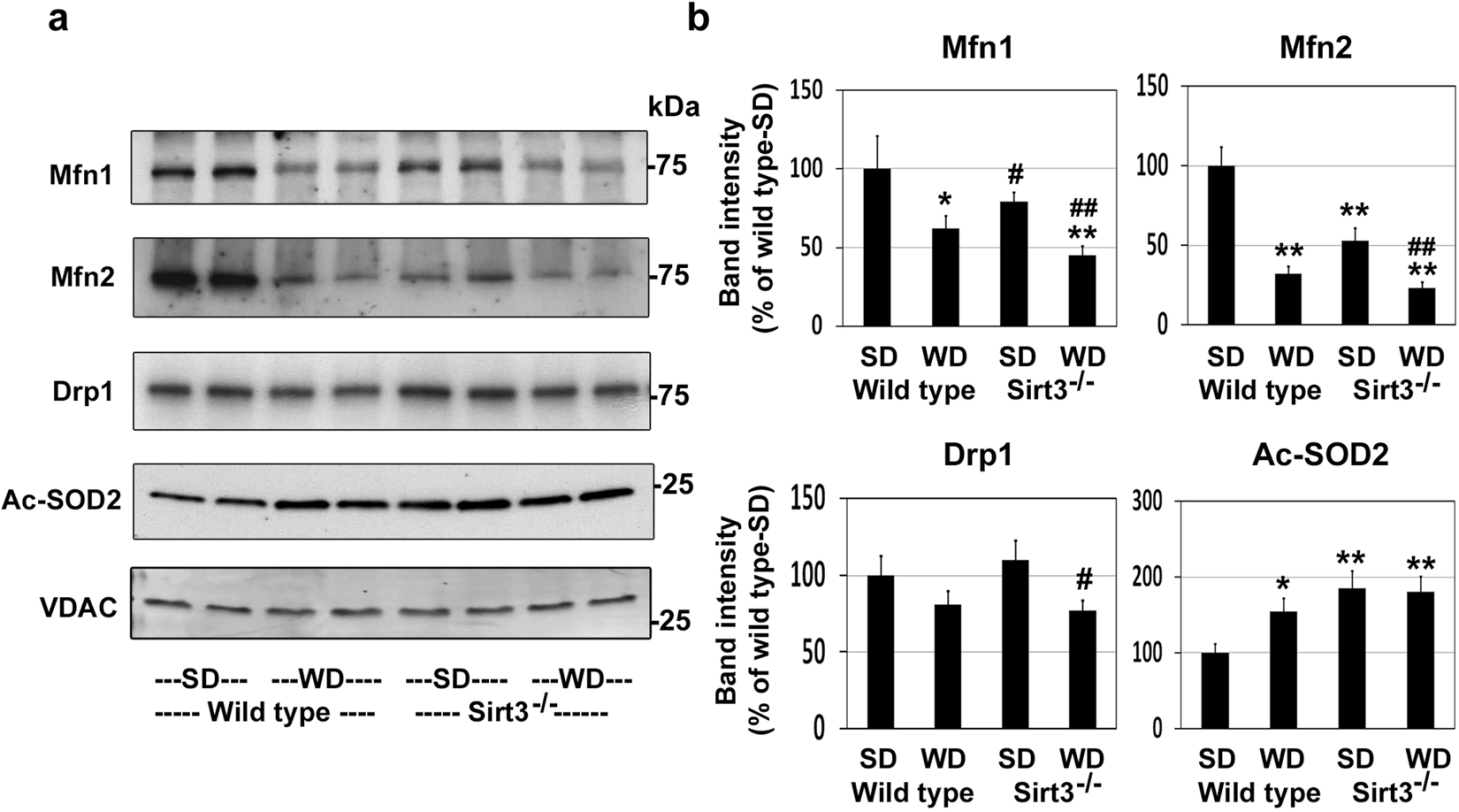
Effects of western diet feeding and Sirt3 deletion on mitochondrial fission and fusion proteins. (**a**) Western blot analysis was performed with mitochondrial extracts of the brain samples from the mice described in Fig. 2 for the mitochondrial fusion and fission proteins and acetyl SOD2. Protein loading was checked by stripping and reprobing the membrane for VDAC. Representative blots of two brain sample from each group are shown. (**b**) Densitometric analysis of band intensity for each blot was corrected for VDAC levels. SD; standard diet and WD; western diet. The results are shown for mean ± SE of 6 samples, as percent of wild type control on standard diet. ^#^*P* < 0.05; *P < 0.01; **P < 0.001 vs wild type-SD. ^##^*P* < 0.01 vs Sirt3^−/−^ mice-SD.

Mitochondrial dynamics involving fission and fusion play significant roles in the regulation of mitochondrial function in response to stress. Mitofusins, Mfn1 and Mfn2, coordinately control mitochondrial fusion event. We observed 38% and 68% lower levels of Mfn1 and Mfn2 respectively, following western diet-feeding in wild type mice. Sirt3 deletion resulted in 25% lower Mfn1 levels and 47% lower Mfn2 levels (Fig. 4a,b). The combination showed maximum lower levels in both mitofusins. In the case of Drp1, mitochondrial fission protein, modestly lower levels (29%) were observed, only with the combination of Sirt3 deletion and western diet feeding (Fig. 4a,b). SIRT3 increases the activity of mitochondrial antioxidant enzyme, superoxide dismutase (SOD2), by deacetylation. We observed the levels of acetyl SOD2 to be 55% higher, following western diet feeding and 85% higher by Sirt3 deletion (Fig. 4a,b). These findings suggest that in addition to metabolic dysregulation, MetS affects mitochondrial dynamics and antioxidant defense in the brain.

### Impaired brain mitochondrial respiration in western diet-fed Sirt3^−/−^ mice

**Figure 5:**
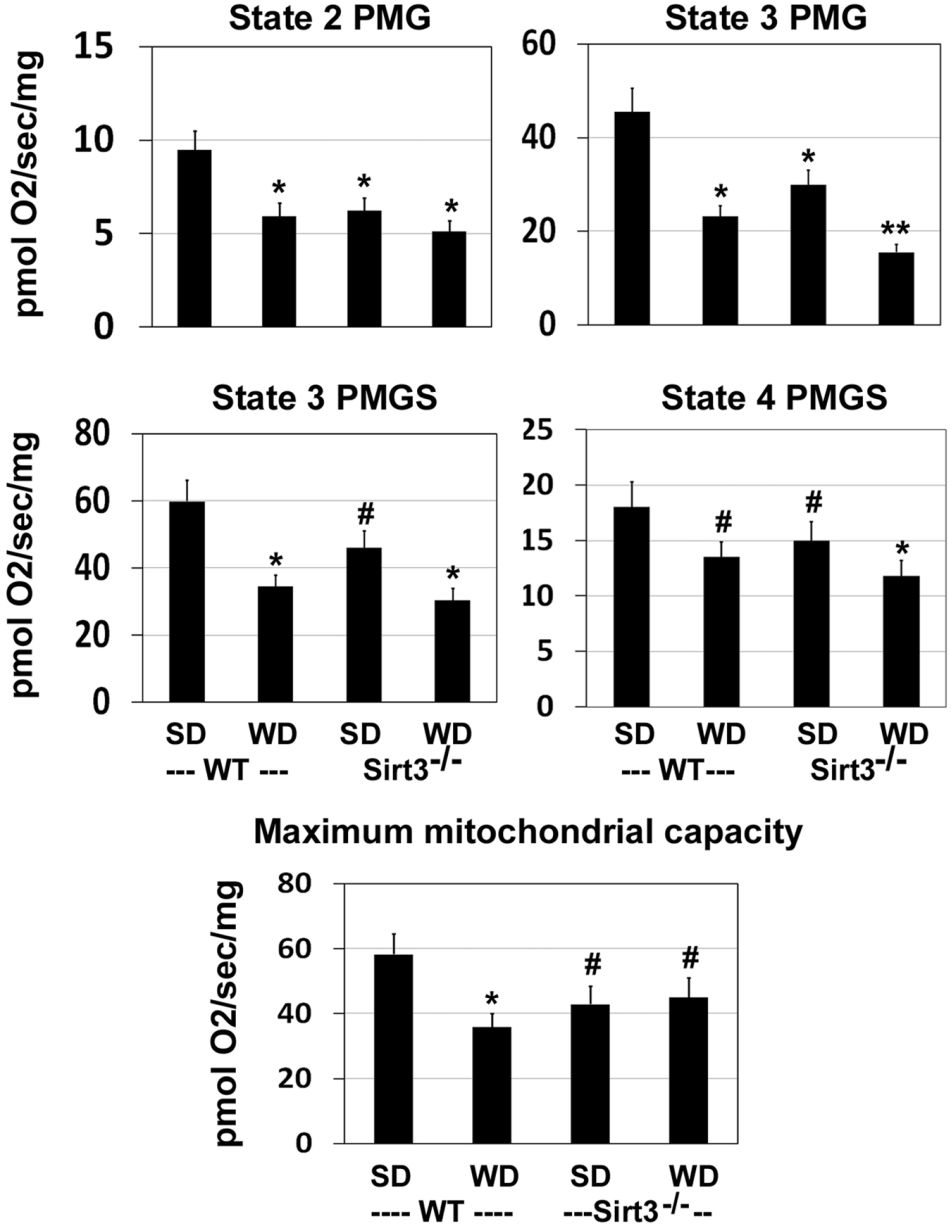
Impairment of brain mitochondrial respiration by western diet feeding and Sirt3 deletion. Respiration rates are presented for the brain cortex samples from wild type (WT) and Sirt3^−/−^ mice on standard (SD) and western diet (WD) at multiple states with the substrates, pyruvate (P) malate (M), glutamate (G) and succinate (S). ^#^*P* < 0.05; *P < 0.01; **P < 0.001 vs wild type-SD.

**Figure 6:**
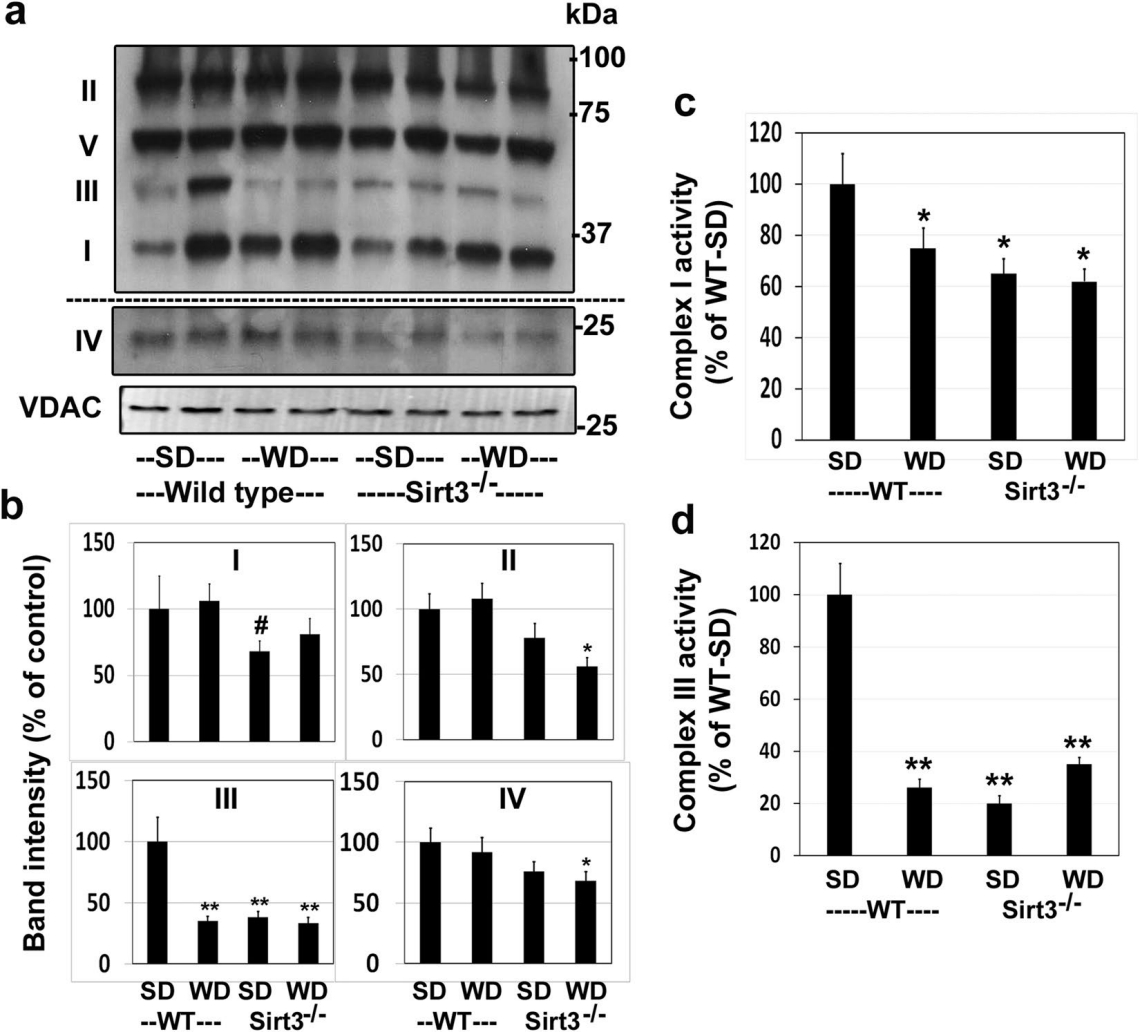
Downregulation of mitochondrial respiration complexes. (**a**) Western blotting with the mitochondrial extracts were performed to determine the levels of mitochondrial respiration complexes I-V. Complex IV band is from the same scan after high exposure for better clarity. (**b**) Band intensities were determined by scanning and corrected for the levels of mitochondrial VDAC. (**c**) Complex I and (**d**) Complex III activities were measured with the mitochondrial lysates. ^#^*P* < 0.05; *P < 0.01; **P < 0.001 vs Standard diet-fed wild type mice.

To determine if downregulation of mitochondrial proteins resulted in decreased mitochondrial function in the brain, we performed respiration studies, using Oroboros Oxygraph-2k, with substrates, pyruvate (P), malate (M), glutamate (G), and succinate (S). Respiration in response to PMG, revealed significantly lower respiration state 2 (ADP-independent), state 3 (ADP-dependent), and state 4 (ADP-independent) following western diet feeding as well as Sirt3 deletion (Fig. 5). The combination showed a significantly (P < 0.01) greater decrease with respect to state 3 (PMG) and state 4 (PMGS). The probable cause of impaired mitochondrial respiration could be the fact that many of the components of electron transport chain were hyperacetylated because of Sirt3 deletion and western diet feeding (Table 2). Next, we examined the subunits of mitochondrial respiratory chain complexes. The levels of complex I were lower by 32% in Sirt3^−/−^ mouse brain whereas the diet did not have any effect (Fig. 6a,b). Complex II levels were lower by 44% in western diet-fed Sirt3^−/−^ mice. The levels of complex III showed maximum lowering of 62–67% (P < 0.001) following western diet feeding and Sirt3 deletion. A combination of Sirt3 deletion and western diet alone lowered significantly the levels of complex IV whereas complex V levels remained unaltered in all the four groups. Thus, brain mitochondrial function is impaired in a mouse model of MetS. Lower levels of respiratory chain complexes were further examined by assaying the enzyme activities for selected complexes. Complex I activity in the brain mitochondrial fractions was lower by 25% in wild type mice, following western diet feeding. In the case of Sirt3^−/−^ mice, the activities were 35% and 38% lower with standard and western diets respectively; (Fig. 6c). Much lower (65–80%) complex III activities were observed because of western diet-feeding as well as Sirt3 deletion, compared to standard diet-fed wild type mice (Fig. 6d). The changes in these complex activities paralleled their corresponding protein levels (Fig. 6a).

### Inflammasome formation in Sirt3^−/−^ mouse brain

**Figure 7:**
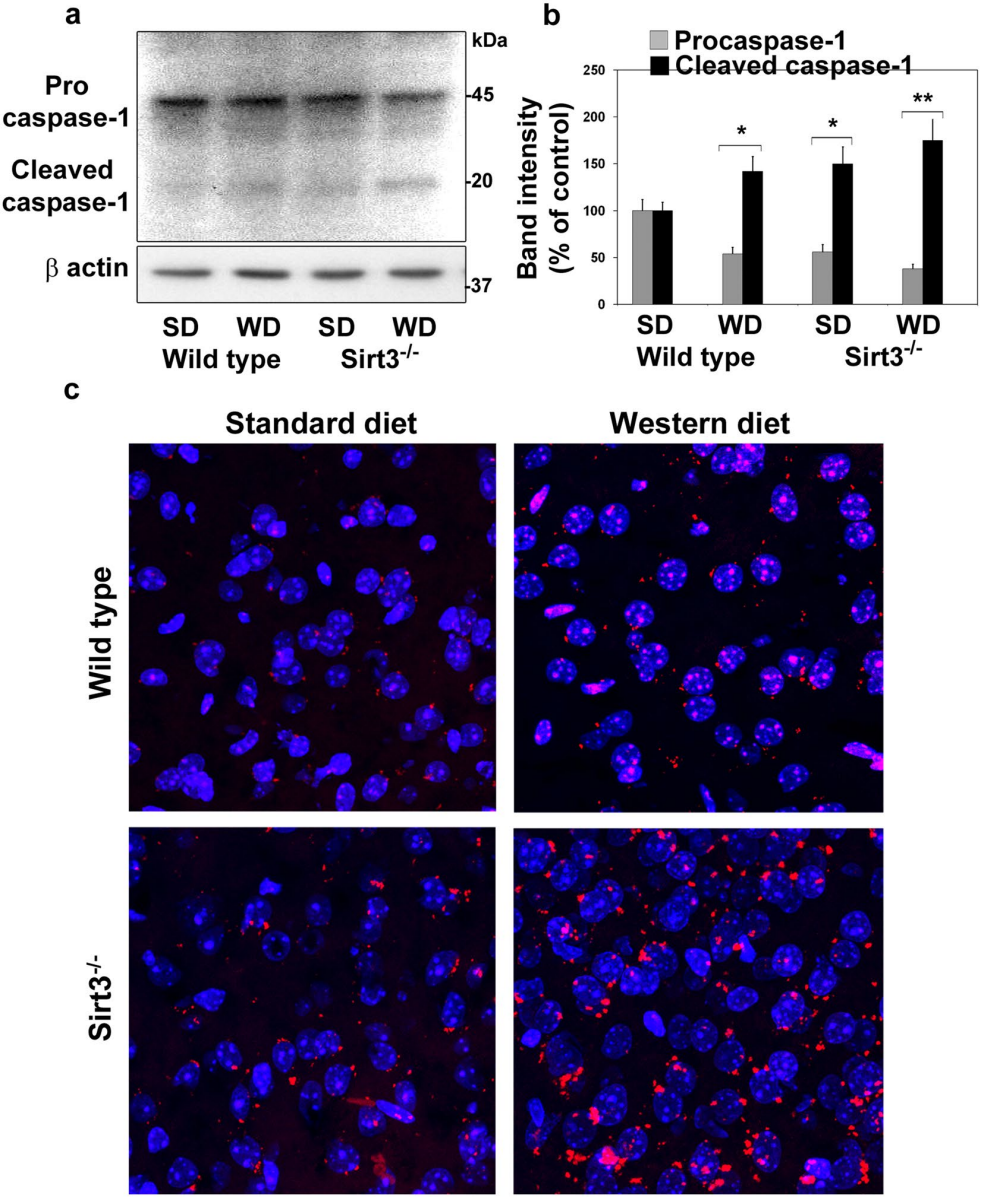
Inflammasome formation in the brain of western diet-fed Sirt3^−/−^ mice. (**a**) Inflammasome formation in the mouse brain samples were examined by western blot analysis of pro and cleaved caspase-1. (**b**) The bands were scanned and corrected for the levels of β actin. *P < 0.01; **P < 0.001 vs Standard diet-fed wild type mice. (**c**) Inflammasome assembly with NLRP3 and caspase-1 was determined by proximity ligation assay and visualized as red dots in red channel. The nuclei were visualized by staining with DAPI (blue).

Inflammasome, a multiprotein complex, consisting of NLRP3, ASC and procaspase is generated in response to infection, cellular damage and metabolic dysregulation^29^. Inflammasome formation leads to the activation of caspase-1 by the proteolytic cleavage of procaspase-1, followed by the release of IL-β and IL-18^30^. Mitochondrial injury is known to induce inflammasome formation, an early triggering step in the inflammatory pathway^26,27^. We tested this link, specifically in the context of Sirt3 deletion-mediated downregulation of mitochondrial proteins. We detected the cleavage of procaspase-1 as shown by 45% lower procaspase-1 levels and 42–50% higher levels of cleaved caspase-1, following western diet feeding and Sirt3 deletion (Fig. 7a,b). Maximum caspase-1 activation was observed with the combination of diet and Sirt3 silencing. To further confirm the assembly of inflammasomes in the brain of a mouse model of MetS, we employed proximity ligation assay. This method detects colocalization of two proteins involving even weak or transient interactions that might not survive the co-IP manipulations. We could visualize the assembly of NLRP3 and caspase-1 as distinct fluorescent dots in the Texas red channel and analyzed by confocal microscopy (Fig. 7c). The inflammasome staining intensities were quantitated at multiple random regions. The quantitation revealed 126% and 82% higher red fluorescence intensity, following western diet and Sirt3 deletion respectively whereas the combination showed 265% higher inflammasome signal. Thus, we could confirm the formation of inflammasomes in the brain during MetS by two methods.

### Diet and Sirt3 deletion-induced microgliosis and markers of neuroinflammation

**Figure 8:**
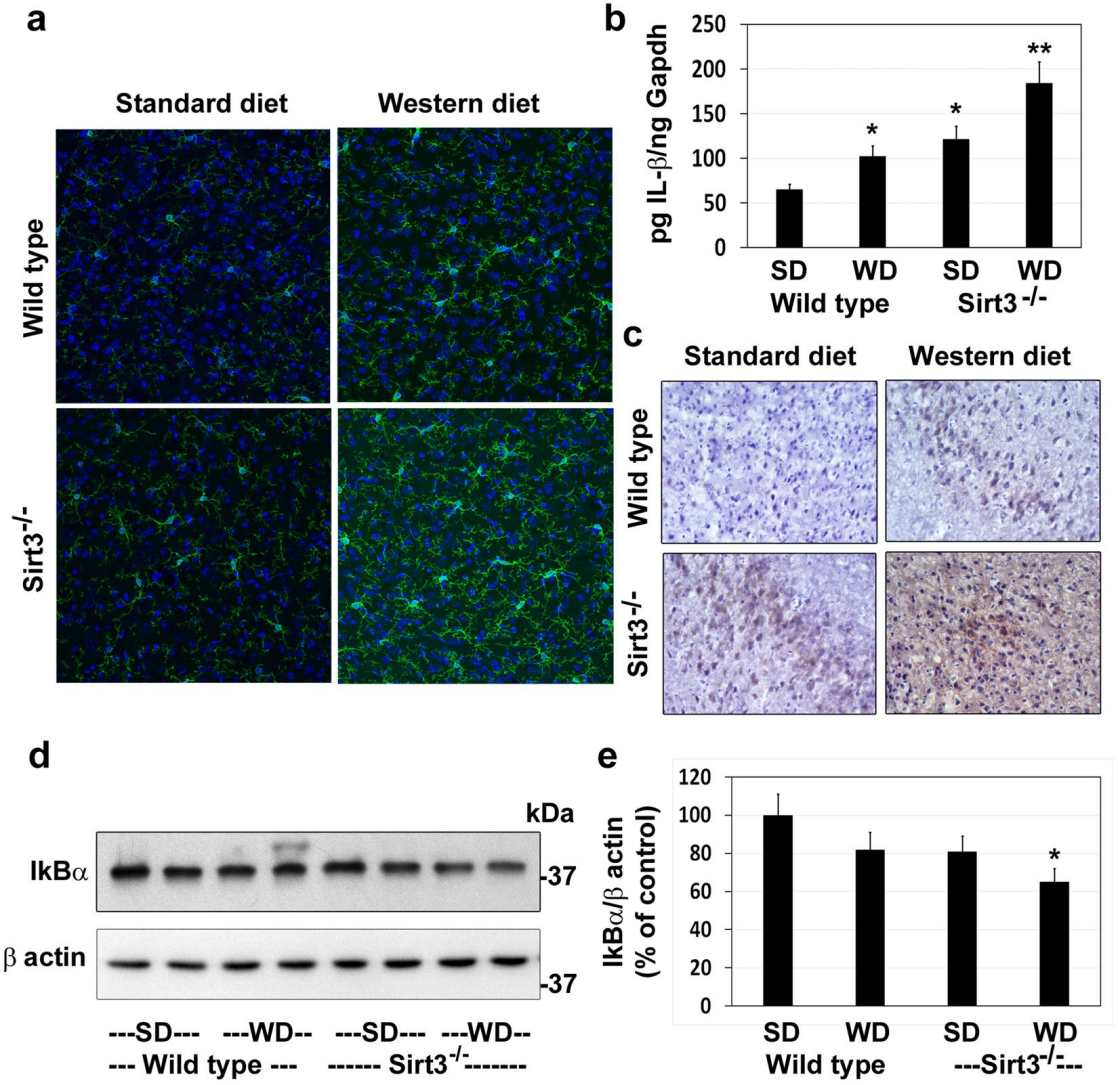
Microgliosis and markers of neuroinflammation in the brain of western diet-fed Sirt3^−/−^ mice. Wild type and Sirt3^−/−^ mice were fed on standard (SD) and western diet (WD) for four months. (**a**) Brain samples were subjected to immunofluorescent staining of Iba1 with FITC (green) for microglia. The nuclei were stained with DAPI (blue). Representative images, captured at 400X magnification in a Leica confocal microscope, are presented. (**b**) The mRNA of IL-1β was determined by real-time PCR in an ABI Prism 7700 sequence detector (Applied Biosystems, Foster City, CA). (**c**) Immunohistochemical analysis of IL-1β was performed with the mouse brain samples. Representative images are presented. (**d**) Western blot analysis of IkBα was performed with the mouse brain samples. The blots were reprobed with β actin antibody. (**e**) The band intensity of IkBα was quantitated by densitometric scanning and corrected for the levels of β actin. The values are mean ± SE of six observations. *P < 0.01; **P < 0.001 vs wild type mice on standard diet.

Circulating CRP levels, an inflammatory marker in plasma, were elevated in western diet-fed and Sirt3-silenced mice (Fig. 1). To further examine other markers of central inflammation, we immunostained the brain sections for Iba1, a marker for microglia, the resident immune cells of the brain. Modest microglial proliferation was observed in western diet-fed wild type mice and in Sirt3^−/−^ mice on standard diet (Fig. 8a). The combination of Sirt3 deletion and calorie overload resulted in significant microgliosis, a marker for neuroinflammation. IL-1β expression in the brain samples followed a similar pattern at the mRNA (Fig. 8b) and protein levels (Fig. 8c). The transcription factor NF-kB plays a key role in inducing the expression of inflammatory mediators. NF-kB is sequestered in the cytosol by the inhibitory protein IkBα. Following the proteosomal degradation of IkB, NF-kB is translocated to the nucleus. Significantly lower IkBα levels were observed in the brain samples of western diet-fed Sirt3^−/−^ mice, suggesting the activation of the inflammatory transcription factor, NF-kB (Fig. 8d,e). Overall, these findings suggest that MetS may induce central inflammation in addition to peripheral inflammation.

## Discussion

While lysine acetylation of histones and its effects on gene expression have been studied extensively, lysine acetylation of non-histone proteins, especially in mitochondria, is a topic of recent interest. This posttranslational modification which decreases the function of target proteins is a key regulatory mechanism. Hyperacetylation of mitochondrial proteins, following global Sirt3 deletion in mice, leads to mitochondrial dysfunction. Previous studies have reported metabolic dysregulation and inflammation in the peripheral tissues of Sirt3^−/−^ mice^8,9,37^. We present here the following new findings in the brain of western diet-fed Sirt3^−/−^ mice: (i) Downregulation of critical mitochondrial proteins in four major pathways of oxidative phosphorylation, fatty acid oxidation, TCA cycle and antioxidant defense (Tables 1–3), (ii) Decreased mitochondrial function as shown by respiration studies (Fig. 5) and (iii) Inflammasome formation which triggers the inflammatory pathway (Fig. 7) and proliferation of microglia (Fig. 8). Thus, we demonstrate mitochondrial protein hyperacetylation, mitochondrial dysfunction and inflammation formation in the brain of a mouse model for MetS. Given that mitochondrial injury is an important cause of inflammasome formation our findings suggest that these two pathways may be linked.

MetS is a combination of five risk factors, including abdominal obesity, hypertriglyceridemia, insulin resistance, high blood pressure and low levels of good cholesterol (HDL). Presence of any three of these five is generally considered to suggest MetS. Around 35% of adults in the US have MetS^38^. We observed higher weight gain, hyperinsulinemia hypertriglyceridemia and elevated circulating CRP in western diet-fed Sirt3^−/−^ mice (Fig. 1). MetS, a precondition for obesity, diabetes, hypertension and cardiovascular diseases (CVD), is caused by genetic factors and life style changes. Single nucleotide polymorphism of human SIRT3 is associated with susceptibility to MetS^11^. SIRT3 is transcriptionally upregulated by calorie restriction and exercise^12,39^. Conversely, chronic high-fat diet feeding lowers SIRT3 levels and depletes NAD^+^, a cofactor for SIRT3 activity^11^. Thus, SIRT3 downregulation is a critical upstream event in the path leading to MetS. While the SIRT3’s functions in peripheral metabolic regulation have been extensively studied^5,9,36,37^, SIRT3’s action in the brain is beginning to emerge^12^. We used global Sirt3^−/−^ mice in this study because whole body metabolic dysregulation is expected to induce metabolic stress in the brain. Furthermore, tissue-specific Sirt3 silencing does not lead to dramatic metabolic impairment^10^. Previous studies have suggested that the CNS effects of MetS may be secondary to systemic changes. We present here findings to show that MetS may exert direct effects in the brain mitochondria by downregulation of metabolic enzymes.

Targets of SIRT3 include electron transport chain complexes and enzymes of intermediary metabolism and antioxidant defense^40^. Although a previous study had examined mitochondrial acetylome in Sirt3^−/−^ mice in multiple tissues, including brain^16^, we have performed this analysis for the first time with a combination of Sirt3 deletion and high-fat western diet feeding, a model for MetS. We identified 103 brain mitochondrial proteins with significantly higher levels of hyperacetylation. A careful examination of the acetylated sites in these proteins suggested their potential downregulation, based on previous studies in peripheral tissues^15,16,36^. A close look at the list of hyperacetylated proteins reveals that SIRT3 may regulate major pathways in mitochondria. Gene ontology (GO)/pathway analysis further showed the hyperacetylated mitochondrial proteins to be primarily in four major pathways, namely, electron transport chain, fatty acid oxidation, TCA cycle and redox pathway (Tables 1–3). Specifically, in the electron transport chain complex I, seven subunits (Ndufa2, Ndufv2, Ndufs3, Ndufs4, Ndufa7, Ndufa9, and Ndufb11) were hyperacetylated. We did observe impaired mitochondrial respiration in Sirt3^−/−^ mouse brain with further exacerbation after western diet feeding (Fig. 5).

A comparison of acetylome analysis in multiple tissues by a previous study revealed that brain, heart and muscle had greater percentage of hyperacetylated sites when compared to liver and kidney in Sirt3^−/−^ mice^16^. SIRT3 is a key mediator of metabolic coupling between fuel-producing and fuel-consuming tissues. Therefore, global Sirt3 deletion is expected to result in an imbalance in the two processes. SIRT3 is also needed for adaptation to diet through metabolic flexibility in mitochondria. The substrates needed for the brain are largely generated in the peripheral tissues, especially in the liver. The substrate availability for the different brain cell types, each with its preferences, is expected to be affected, following Sirt3 deletion. For example, neurons and quiescent microglia produce energy by oxidative metabolism, involving TCA cycle and ETC, whereas glycolysis is predominant in astrocytes and activated microglia^41,42^. Sirt3 deficiency will impair the interdependent relationship between brain cells for energy substrates. Furthermore, accumulating metabolites are important regulators of innate immunity and inflammation^43^. For example, succinate is known to induce IL-1β by stabilizing HIF-1α^44^. Thus, downregulation of metabolic enzymes in the brain can potentially activate the inflammatory pathway.

In addition to hyperacetylation of proteins in the mitochondria, we observed lower levels of mitochondrial fission and fusion proteins following western diet feeding and Sirt3 deletion. Mitochondrial dynamics represent the remodeling of mitochondrial structure in response to metabolic challenges. Mitochondrial fission and fusion play important roles during metabolic stress^45^. The balance between these processes is critical for mitochondrial energetics. Fusion is regulated by mitofusin proteins (Mfn1 and Mfn2) and short and long isoforms of optic atrophy 1 (Opa1) protein. Mitochondrial fission is regulated by fission 1 (Fis1) protein and dynamin related protein (Drp1). We examined representative members of these pathways and observed their downregulation following Sirt3 deletion and western diet feeding (Fig. 4). Furthermore, SIRT3 is known to increase the activity of mitochondrial antioxidant enzyme, SOD2 by deacetylation. We observed hyperacetylation of SOD2, suggesting its downregulation. These findings suggest that injury response of brain mitochondria is likely to be lower in MetS. It needs to be mentioned here that SIRT3 levels are higher after 4 months of western diet feeding (Fig. 2c) as a compensatory response. A longer duration of calorie excess which is known to lower SIRT3 levels^36^, will likely show more dramatic downregulation of mitochondrial function.

As reported previously, peripheral inflammation was evident from the elevated levels of CRP in circulation (Fig. 1). C-reactive protein is an acute phase protein the levels of which increase during inflammation. It is produced in the liver in response to interleukin-6 secreted by macrophages. The findings in this study suggest a potential link between mitochondrial injury and inflammation. Mitochondria-generated ROS are kept at a low level by a finely regulated system that maintains a balance between production and elimination^46^. The enzymes of redox pathway, including SOD2, are downregulated in Sirt3^−/−^ mouse brain (Table 3 and Fig. 4). Therefore, SIRT3 deficiency may cause an imbalance in ROS production and elimination. Furthermore, several markers of mitochondrial dysfunction are observed, following Sirt3 deletion. Taken together, these changes can be triggers of inflammasome formation. Although mitochondria-generated damage-associated molecular patterns (DAMPs) have been shown to trigger inflammasome formation^26,27,47^, we have identified an upstream event, namely mitochondrial protein hyperacetylation, as possible cause of inflammasome formation. While examining the link between peripheral and central inflammation, previous studies have focused on the mechanism that cytokines generated in the periphery cross BBB and induce secondary inflammation in the brain^48,49^. We propose a new mechanism by which CNS inflammation can be triggered through MetS-induced mitochondrial injury in the brain, leading to inflammasome formation. However, a future study is needed to determine if Sirt3 overexpressing mice are resistant to western diet-induced inflammasome formation so that a causal relationship can be established.

We observed proliferation of microglia in the brain of Sirt3^−/−^ mice, especially after western diet feeding. Microglia constitute 5–10% of the brain cells with region-specific variations. Microglial synaptic pruning plays a key role in the establishment of neuronal network^50^. Microglia serve the housekeeper function by constantly monitoring the brain environment. As sensomes, microglia respond to metabolic stress in the brain, modulate their receptor signatures and change their phenotype. Although microglia have protective actions in Alzheimer’s disease (AD) by reducing Aβ load^51^, their sustained activation during chronic inflammation is an important cause of neurodegeneration^52,53^. A major portion of the neuronal energy consumption takes place at the synapses^54^. Microglia are dynamically in touch with the synapses. Therefore, metabolic stress at the synapses will affect microglial proliferation and behavior. SIRT3 deficiency is expected to induce metabolic stress within microglia as well. Because of their constant patrolling function, microglia are in constant demand for ATP^42^. Quiescent microglia utilize glucose by oxidative phosphorylation whereas glycolysis is the preferred pathway after reactivation^42^. This shift, known as Warburg effect, originally observed in cancer cells has been reported in macrophages as well^43^. Therefore, downregulation of TCA cycle and electron transport chain will lead to dysregulation of these bioenergetic pathways.

Glucose-centric management of diabetes, caused by MetS, has generally focused on the traditional complications, including diabetic retinopathy, peripheral neuropathy, nephropathy, and cardiovascular diseases. The CNS effects of chronic diabetes are beginning to be recognized^55,56^. Studies have also reported that mid-life obesity is an important cause of late-life dementia^57,58^. Furthermore, Alzheimer’s disease, the common cause of dementia, is preceded by a long cellular phase which could also take place around mid-life^59^. Brain mitochondrial dysfunction and neuroinflammation, caused by MetS in mid-life may accelerate the path to dementia in individuals susceptible for AD.

## Methods

### Animals and treatment

Animal care and the experimental procedures involving animals were approved by Institutional Animal Care and Use Committee at the Rocky Mountain Regional Veteran Administration Medical Center. Wild type and global Sirt3^−/−^ mice in 129Sv background were purchased from Jackson Laboratory (Bar Harbor, ME). Two-month-old male mice (6/group) were fed ad libitum, a standard diet (TD.2018 Envigo, Indianapolis) or a calorie rich western diet (TD.88137, Envigo, Indianapolis). Standard diet consisted of 18.6% protein, 44.2% carbohydrate and 6.2% fat by wt which generate 24% kcal, 58% kcal and 18% kcal, respectively. The composition of western diet was 17.3% protein (15.2% kcal), 48.5% carbohydrate (42.7% kcal) and 21.2% fat (42% kcal). After the feeding the mice with respective diets for four months, they were sacrificed, after isoflurane exposure. Blood was collected by cardiac puncture for the separation of plasma. Following perfusion with PBS, one half of the brain was immersed in the fixative solution, cryopreserved in 30% sucrose solution overnight and then embedded in OCT. After saving a small portion of the fresh cortex samples for mitochondrial respiration studies, the rest of the other brain hemisphere was snap frozen for western blot and RNA analyses. All methods described in this section were performed in accordance with the relevant guidelines and regulations.

### Plasma assays

Plasma insulin levels were determined, using a kit from ALPCO, Salem, NH (Cat. #80-INSMS-E01). Plasma triglyceride levels were measured, using a colorimetric kit from Abcam, Cambridge, MA (Cat. #ab65336). C-reactive protein (CRP) levels in plasma were measured by ELISA, using a kit from R&D Systems, Minneapolis, MN (Cat. #MLB00C).

### Western blot analysis

The brain tissues were lysed with mammalian protein extraction buffer (Pierce, Rockford, IL). Lysates of brain tissue or isolated mitochondria from four groups of mice were analyzed by western blot analysis as previously described^60^. Briefly, samples with equal amount of protein (30–50 µg) were resolved on 7.5% or 12% tris glycine gels by SDS-PAGE. Separated proteins were transferred onto polyvinylidene difluoride (PVDF) membrane. The membranes were blocked in 5% nonfat milk for 1 h at RT and then incubated with specific primary antibody (1:1000) at 4 °C for overnight. The antibodies against acetyl lysine (Cat # 9441), Sirt1 (Cat # 9475), Sirt3 (Cat # 5490), Sirt5 (Cat # 8782), Drp1 (Cat # 14647), Ac-p65 (#12629), p65 (#3034) and β actin (Cat # 4967) were from Cell Signaling Technology (CST, Danvers, MA). Anti-SOD2 (acetyl K68; Cat # ab137037), anti-Mfn1 (Cat # ab57602) and anti-Mfn2 (Cat # ab124773) and OXPHOS antibody cocktail (Cat # ab110412) were from AbCam (Cambridge, MA). After washing in TBST, the membranes were exposed to secondary antibodies conjugated to alkaline phosphatase and the signals were developed with CDP-Star reagent (Sigma Aldrich-St Louis, MO). The band intensities were measured using Fluor S MultiImager and Quantity One software from Bio-Rad and corrected for the levels of β actin.

### Tryptic digestion of mitochondrial proteins and enrichment of acetylated peptides

Mitochondria were isolated from the mouse brain samples by differential centrifugation and the lysates were subjected to acetylome analysis by the procedures described before^61^ with modifications. Briefly, mitochondria-enriched fractions with equal protein content (2 mg) were trypsinized and subjected to acetyl-lysine immunocapture using PTMScan Acetyl-Lysine Motif kit from CST (Cat #13416). Doping with an acetylated BSA internal standard was done for IP quality control and sample-to-sample normalization for quantification. Peptides were purified with a 0.7 ml C18 Sep- Pak column (Waters Corporation, Milford, MA) and the eluted peptides were lyophilized. Acetylated peptides were enriched using the anti-acetyl immunoaffinity beads provided in the kit and resuspended in 3% acetonitrile in 0.1% formic acid for MS analysis.

### LC-MS/MS identification of acetylated brain mitochondrial proteins

The enriched acetyl-Lys peptides were chromatographically resolved using Acclaim PepMAP RSLC reverse phase nano column (Thermo scientific). Data collection was on a 6550 Q-TOF equipped with a nano source (Agilent) operated using intensity-dependent CID MS/MS to generate peptide IDs. MS/MS data were collected in positive ion polarity over mass ranges 290–1700 m/z at a scan rate of 10 spectra/sec for MS scans and mass ranges 50–1700 m/z at a scan rate of 3 spectra/sec for MSMS scans. SpectrumMill software (Agilent) was used to extract, search, and summarize peptide identity results. A minimum peptide score of 8, scored peak intensity of 505 and protein score of 10 were used as cut offs for the generation of an AMRT library. Data analysis: MS Quant data was extracted and aligned using a recursive workflow with Profinder V.B.08.00 software (Agilent). The extracted compound list was exported to Mass Profiler Professional V.14.8 (Agilent, MPP). The score is based on how the quality of the mass, isotope abundances and isotope spacing of compounds found in each sample match to a targeted chemical formula within a specified retention time window. Final extraction and alignment results were exported to MPP for data analysis. Peptides were annotated using ID Browser software (Agilent) by matching the mass and retention time from aligned experimental data to peptide hits in the AMRT library generated from MS/MS data. At the peptide level a combination of a fold change of >2.0 and P < 0.05 was considered significant. For overall protein acetylation changes, the raw abundance of all acetyl peptides regardless of significance were rolled up into total protein abundance for each individual protein. At the protein level if the fold change was >1.5 and >30% of all peptides, identified for that protein were significant, then the protein was considered significant. Statistical analysis was performed by 2-way ANOVA or moderated T-Test. Venn diagram and Volcano plots were obtained to determine the effects of diet and genotype.

### Measurement of mitochondrial oxygen consumption in brain tissue

Cortical tissues were hand homogenized in ice-cold mitochondrial isolation buffer (MIB: 70 mM sucrose, 210 mM mannitol, 5 mM HEPES, 1 mM EGTA and 0.5% fatty acid free BSA, pH 7.2) with 20 strokes. The homogenate was centrifuged at 500 g for 10 min at 4 °C. The supernatant was used for the measurement of oxygen consumption at 37 °C, using Oroboros Oxygraph-2k (O2K; Oroboros Instrument Corp, Innsburk, Austria). Homogenates containing 4 mg of cortical tissues were re-suspended in mitochondrial respiration buffer [MiR06 (0.5 mM EGTA, 3 mM magnesium chloride, 60 mM K-lactobionate, 20 mM taurine, 10 mM potassium phosphate, 20 mM HEPES, 110 mM sucrose, 1 g/L bovine serum albumin, 280 u/mL catalase, pH 7.1)]. Substrates and inhibitors were added to assess respiration at several states. Rates were measured with the final concentration of 5 mM pyruvate + 2 mM malate + 10 mM glutamate (PMG) + 2 mM adenosine diphosphate (ADP) + 6 mM succinate (S), 10 µg/mL oligomycin, and 0.5 µM stepwise titration of carbonyl cyanide 4-(trifluoromethoxy) phenylhydrazone added until maximal uncoupling (uncoupled state).

### Assay of mitochondrial complex I and III

The brain cortex samples were homogenized with a Dounce tissue homogenizer in mitochondrial isolation buffer (70 mM sucrose, 210 mM mannitol, 5 mM Tris-HCl, 1 mM EDTA; pH 7.4), and suspensions were centrifuged at 800 g (4 °C) for 10 min. Further, supernatants were centrifuged at 8,000 g (4 °C) for 10 min, and pellets were washed with mitochondrial isolation buffer. Complex I (NADH:ubiquinone oxidoreductase) activity was measured in mitochondrial fractions as described previously by Spinazzi *et al*.^62^ with minor modification using BioTek Synergy H1 microplate reader. Briefly, mitochondrial lysates were added in the assay buffer [Kpi (0.5 M), NADH (2 mM), KCN (25 mM), antimycin (1 mg/ml) and defatted BSA (100 mg/ml)] and the reaction was started by the addition of the electron acceptor (ubiquinone-1,5 mM). The oxidation of NADH was recorded every 15 sec for 3 min at 340 nm. Next, activity was further measured following the addition of rotenone (10 µM) every15 sec for 3 min at 340 nm. Differences between the rate of oxidation, before and after the addition of rotenone, was used to calculate the complex I enzyme activity. Freshly isolated mitochondrial fractions were used to measure the kinetics of complex III activity using the kit from BioVision (# K520, Milpitas, CA). Enzyme activity was determined as a change in the optical density/30 sec/µg mitochondrial protein for up to 10 min at 550 nm wavelength.

### Immunohistochemical analysis

The brain tissues were embedded in OCT and the sections (10 µM) were quenched with 0.9% H_2_O_2_ in 0.02% Triton-X100 in TBS, at RT for 30 min. After blocking in normal goat serum (1.26%) in 0.3% Triton-X-100 in TBS (TBST) for 1 h at RT, the slides were incubated with IL-1β antibody (Cat # ab9722; 1:500 dilution) overnight at 4 °C in a humidified chamber. After washing and incubation with biotinylated secondary antibody (1:1000) for 1 h at RT and with ABC solution (ABC Elite kit, Vector Laboratories, Burlingame, CA) for 2 h at RT, the color was developed in DAB/H_2_O_2_ solution (Cat # SK-4100; Vector Laboratories). The sections were counter-stained with Hematoxylin, dehydrated through series of ethanol, cleared in xylene and coverslip was placed with paramount mounting medium. Images were captured at 400X magnification in Olympus BX51 microscope.

### Immunofluorescence staining

The frozen brain sections (10 µm) were permeabilized and blocked as described above. After washing in TBS, sections were incubated with primary antibody against Iba1 (Cat # 019-19741, Wako, Richmond, VA; 1:200 dilution) in TBST overnight at 4 °C in humidified chamber and subsequently then washed in TBST. The slides were incubated with FITC-conjugated secondary antibody and DAPI in the dark for 2 h at RT, followed by washes in TBST. The sections were mounted with Prolong®Gold antifade reagent (source) and covered with coverslips. The images were captured in a Leica confocal microscope. Fluorescence intensity was quantitated using LAS X software.

### Proximity Ligation Assay

Inflammasome assembly was examined by proximity ligation assay, using DuoLink *In Situ* Orange Starter Kit Mouse/Rabbit (Cat # DU092102; Sigma-Aldrich, St Louis, MO). Briefly, the brain sections were blocked with DuoLink blocking buffer for 30 min at 37 °C and incubated with primary antibodies against caspase-1 (anti-mouse; Cat # SC56036; Santa Cruz) and NLRP3 (anti rabbit; Cat # AG-20B-0014, AdipoGen Life Sciences, San Diego, CA) for 1 h, washed and then further incubated for 1 h at 37 °C with species-specific PLA probe under hybridization conditions. Hybridized oligonucleotides were ligated to form a closed circle, which serves as a template for rolling-circle amplification after adding an amplification solution to generate a concatemeric product extending from the oligonucleotide arm of the PLA probe. Fluorescently labelled oligonucleotides were further hybridized to the products generated and the signal was detected as distinct fluorescent dots in the Texas red channel. The images were captured in a Leica confocal microscope.

### Statistical analysis

To identify the statistical significance difference between groups, one-way ANOVA followed by a Bonferroni’s test using Sigma Stat version 3.5 software was employed and two-sided p values of <0.05 were considered significant.

## Electronic supplementary material

Supplementary information

## Data Availability

Additional data sets generated and/or analysed during the current are available from the corresponding author on reasonable request.
