# Supplementary material for: SIRT3 deficiency-induced mitochondrial dysfunction and inflammasome formation in the brain
Source: Sci Rep. 2018 Dec 3;8:17547. doi: 10.1038/s41598-018-35890-7 (PMC6277395; doi:10.1038/s41598-018-35890-7)
Supplement: Supplementary file 1 — Supplementary information [file 41598_2018_35890_MOESM1_ESM.pdf]

SIRT3 deficiency-induced mitochondrial dysfunction and inflammasome formation in the brain

Alpna Tyagi[1,2], Christy Nguyen[1], Thomas Chong[1], Michel Cole[3], Kristofer S Fritz[3], Nichole Reisdorph[3], Leslie Knaub[1,2], Jane EB Reusch[1,2], Subbiah Pugazhenthi*[1,2]

[1]Rocky Mountain Regional VA Medical Center

[2]Department of Medicine, [3]Department of Pharmaceutical Sciences

University of Colorado-Anschutz Medical campus, Aurora, CO, USA

*Address for correspondence:

Subbiah Pugazhenthi, Ph.D.

Rocky Mountain Regional VA Medical Center and

Department of Medicine

University of Colorado; Anschutz Medical Campus

Aurora, CO USA

Phone: 720-857-5629;

Fax: 303-377-5686;

Email: subbiah.pugazhenthi@ucdenver.edu

Running title: Brain mitochondrial dysfunction in Sirt3$^{-/-}$ mice

Key words: Sirt3, western diet, protein lysine acetylation, acetylome analysis, mitochondrial dysfunction, inflammasome, microglia and neuroinflammation

Figure 2a. **VDAC** **kDa**

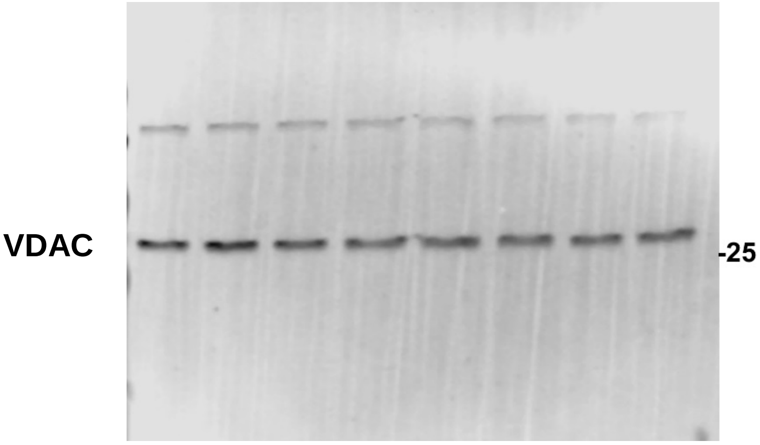

Figure 2c. **SIRT1** **kDa**

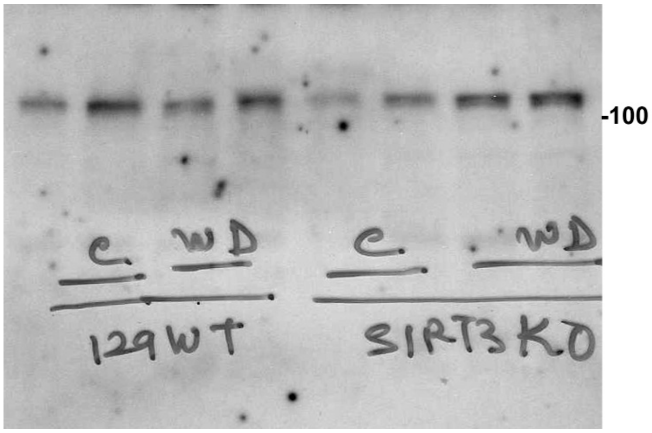

Figure 2c. **SIRT3** **kDa**

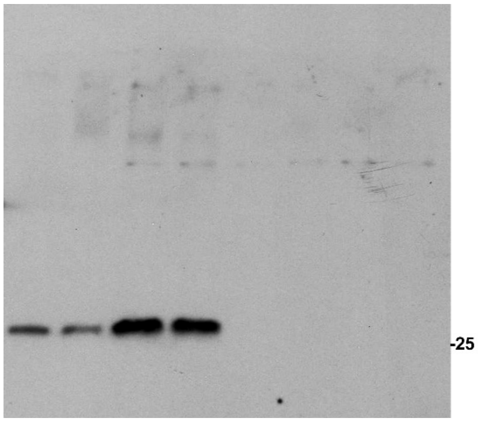

Figure 2c **Ac-p65** **kDa**

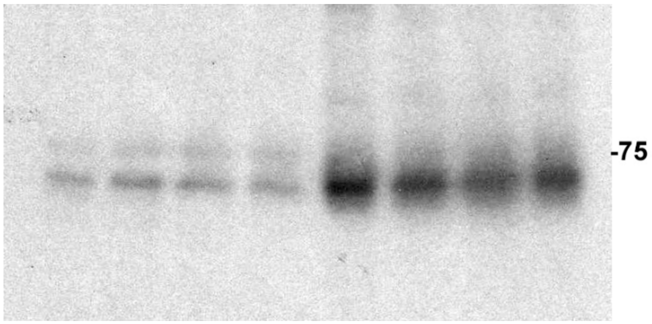

Figure 2c **p65** **kDa**

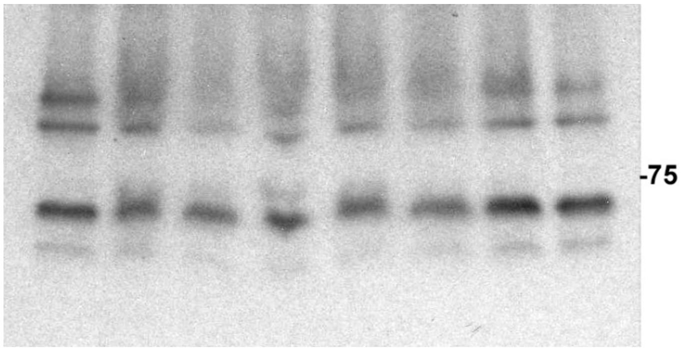

Figure 2c. SIRT5 kDa

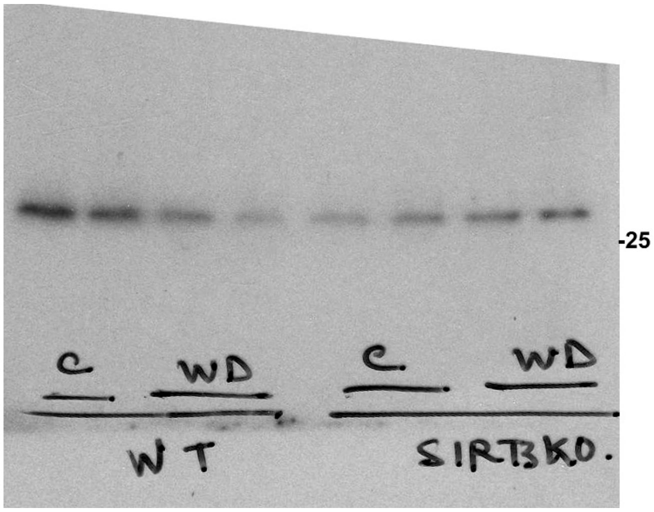

Figure 2c. β actin kDa

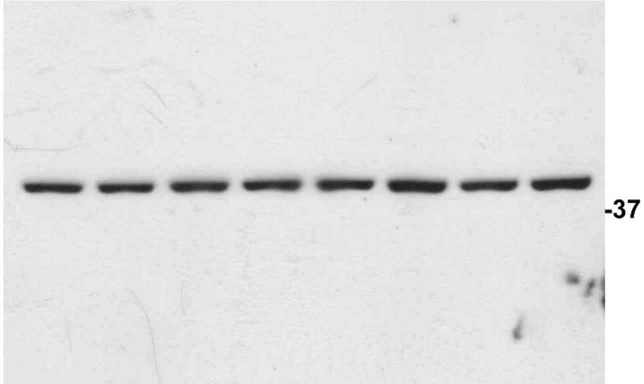

Figure 4a. Mfn1 kDa

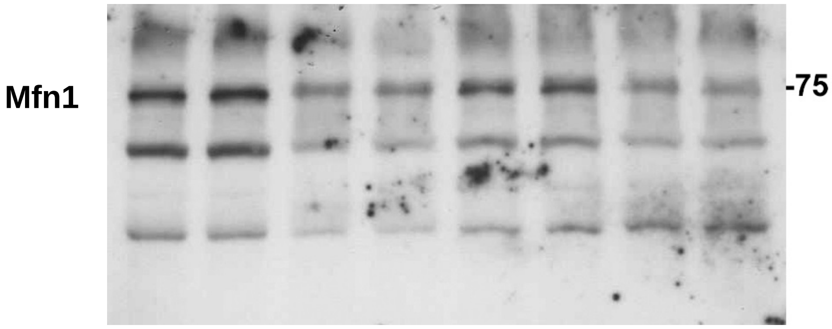

Figure 4a. Mfn2 kDa

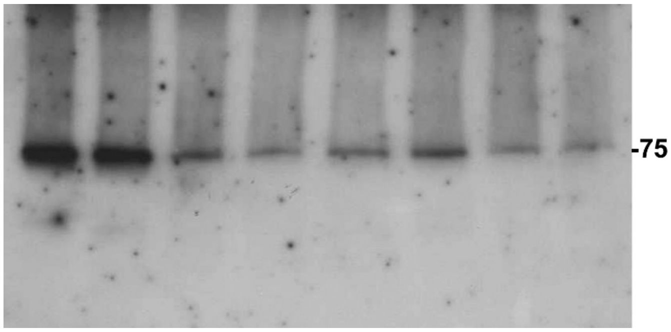

Figure 4a. Drp1 kDa

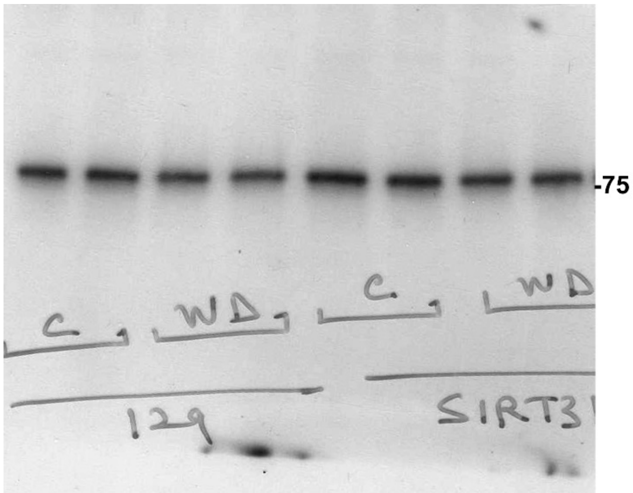

Figure 4a. Ac-SOD2 kDa

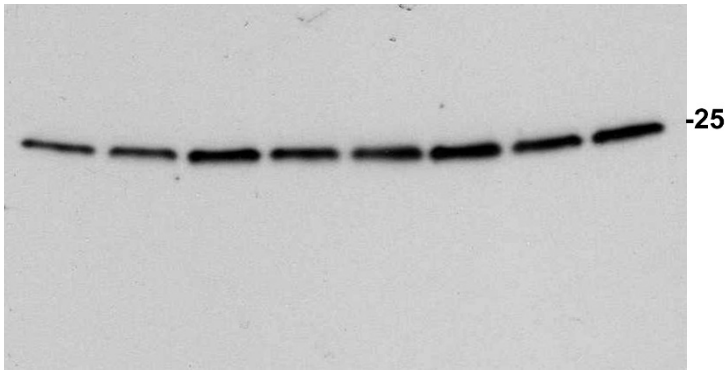

Figure 4a.

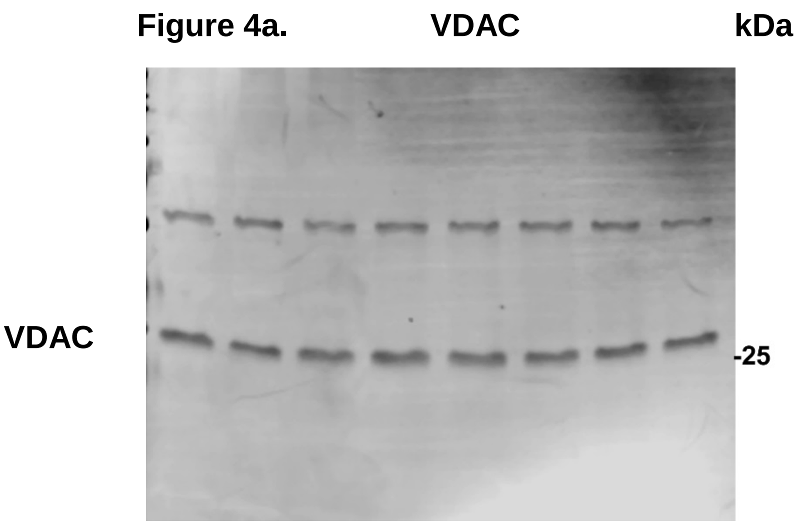

Figure 6a. Mito complex (Light exposure) kDa

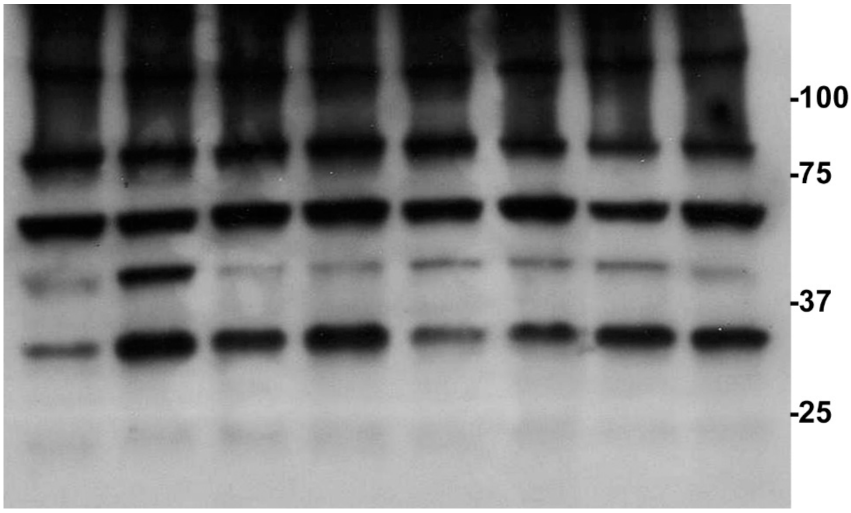

Figure 6a. Mito complex (Dark exposure) kDa

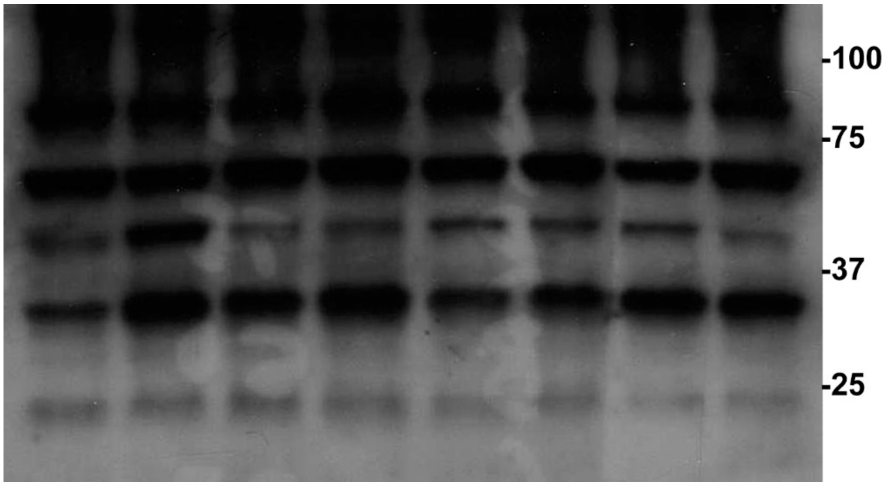

Figure 6a. VDAC kDa

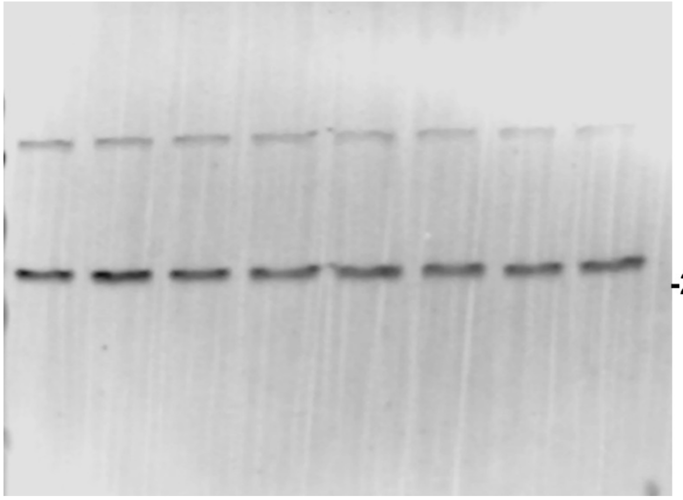

Figure 7a. **β actin** **kDa**

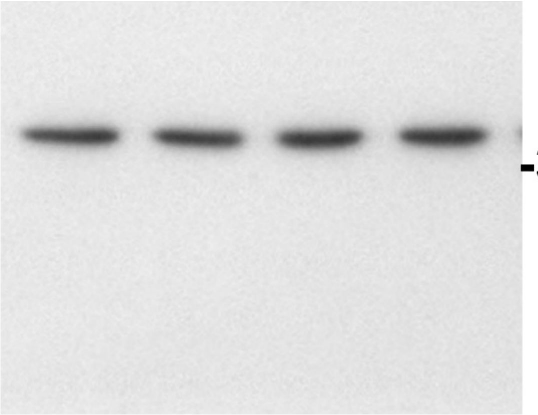

-37

Figure 8d. **IkBα** **kDa**

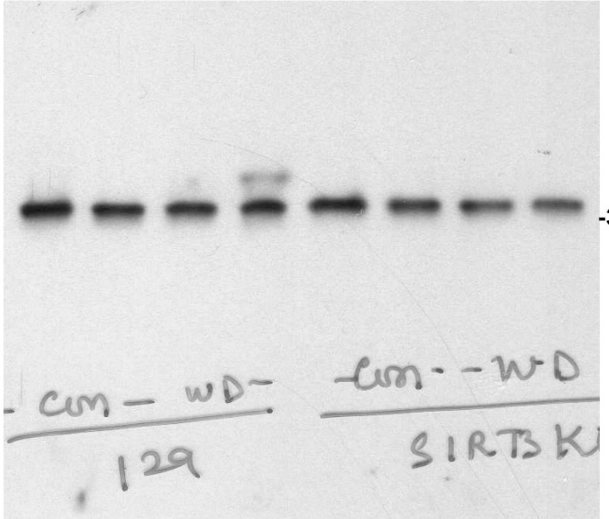

-37

Figure 8d. **β actin** **kDa**

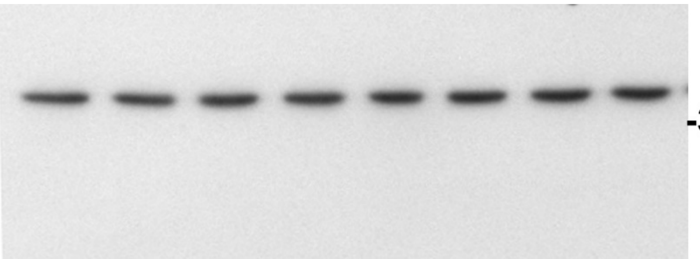

-37
